# The Association of Cell Division Regulated by DicC With the Formation of Viable but Non-culturable *Escherichia coli* O157:H7

**DOI:** 10.3389/fmicb.2019.02850

**Published:** 2019-12-10

**Authors:** Hanxu Pan, Kai Dong, Lei Rao, Liang Zhao, Yongtao Wang, Xiaojun Liao

**Affiliations:** ^1^Beijing Advanced Innovation Center for Food Nutrition and Human Health, College of Food Science and Nutritional Engineering, China Agricultural University, Beijing, China; ^2^Key Laboratory of Fruit and Vegetable Processing, Ministry of Agriculture and Rural Affairs, Beijing, China

**Keywords:** VBNC, DicC, cell division, growth rate, cell morphology

## Abstract

The viable but non-culturable (VBNC) state, in which bacteria fail to grow on routine culture media but are actually alive, has been widely recognized as a strategy adopted by bacteria to cope with stressful environments. However, little is known regarding the molecular mechanism of VBNC formation. Here, we aimed to elucidate the specific roles of cell division regulatory proteins and the cell growth rate during VBNC *Escherichia coli* O157:H7 formation. We have previously found that expression of *dicC* is reduced by 20.08-fold in VBNC *E. coli* O157:H7 compared to non-VBNC cells. Little is known about DicC except that it, along with DicA, appears to act as a regulator of cell division by regulating expression of the cell division inhibitor DicB. First, our results showed that the VBNC cell number increased in the Δ*dicC* mutant as well as the DicA-overexpressing strain but decreased in the DicC-overexpressing strain induced by high-pressure carbon dioxide, acid, and H_2_O_2_. Furthermore, the growth rates of both the DicA-overexpressing strain and the Δ*dicC* mutant were higher than that of the control strain, while DicC-overexpressing strain grew significantly more slowly than the vector strain. The level of the *dicB* gene, regulated by *dicA* and *dicC* and inhibiting cell division, was increased in the DicC-overexpressing strain and decreased in the Δ*dicC* mutant and DicA-overexpressing strain, which was consistent with the growth phenotypes. In addition, the dwarfing cell morphology of the Δ*dicC* mutant and DicA-overexpressing strain were observed by SEM and TEM. Taken together, our study demonstrates that DicC negatively regulates the formation of the VBNC state, and DicA enhances the ability of cells to enter the VBNC state. Besides, the cell growth rate and dwarfing cell morphology may be correlated with the formation of the VBNC state.

## Introduction

The viable but non-culturable (VBNC) state, a unique biological state, is a positive response strategy used by a number of bacteria to cope with different adverse environments (Colwell, 2000; Oliver, 2005b; Orruno et al., 2017). Xu et al. (1982) found that *Escherichia coli* and *Vibrio cholerae* from seawater could not be cultured on their routine medium but possessed metabolic activity. Based on this finding, they proposed the concept of the VBNC state, in which bacteria cannot grow on the routine culture medium but are actually alive. Subsequently, they further proved that these bacteria can recover from the VBNC state and become culturable again when the environment is suitable, which is termed resuscitation (Roszak et al., 1984). A growing number of VBNC microorganisms have been found in various environments, posing a significant risk to public health (Li et al., 2014; Zhao et al., 2017). To date, approximately 110 species of microorganisms, including bacteria and fungi, have been reported to enter the VBNC state when exposed to diverse forms of stress, and these species can be divided into three categories based on their features and functions, namely, microbes associated with food and medical safety, environmental applications and agricultural diseases (Ramamurthy et al., 2014; Ayrapetyan and Oliver, 2016; Ayrapetyan et al., 2018). Thus, VBNC microorganisms are widely distributed in nature, representing a strategy used by microorganisms to survive in different adverse environments.

A range of stressful environmental conditions during food processing and preservation can also induce the entry of many foodborne pathogens into VBNC state, for instance, high-temperature sterilization of milk, chlorination of wastewater, extreme temperatures, ultraviolet (UV) radiation, ultrasound, irradiation, drying, pulsed electric field (PEF), high-pressure carbon dioxide (HPCD) and high-pressure stress, as well as the addition of preservatives and disinfectants (Oliver, 2005a; Zhao et al., 2013, 2017; Zhang et al., 2015). Therefore, the VBNC bacteria should not be neglected. In addition, a series of morphological and physiological changes occur when microorganisms enter the VBNC state. The morphological changes affect mainly cell size, cell wall composition and the cell membrane (Signoretto, 2000; Signoretto et al., 2002; Day and Oliver, 2004; Oliver, 2010; Chen et al., 2018). The physiological features of VBNC cells are mainly characterized by decreased metabolic activity, slow absorption of nutrients, potential pathogenicity, decreased gene expression and protein translation capacity, and increased resistance to antibiotics (Du et al., 2006; Li et al., 2014; Lin et al., 2017).

*Escherichia coli* is one of the most widely studied bacteria in VBNC research, and there are many studies on the conditions associated with induction and recovery of the VBNC state of this species as well as on the formation mechanisms (Ding et al., 2016). In this study, *E. coli* O157:H7 was adopted as the target strain to study the molecular mechanism underlying the formation of the VBNC state. *E. coli* O157:H7, the major pathogenic serotype of enterohemorrhagic *E. coli*, can cause diarrhea, hemorrhagic enteritis, and two serious complications, namely, hemolytic uremic syndrome (HUS) and thrombotic thrombocytopenic purpura, and produce mortality (Karmali et al., 1985; Nataro and Kaper, 1998). Since it was first discovered in 1983, *E. coli* O157:H7 has caused a worldwide epidemic. *E. coli* O157:H7 can enter the VBNC state under a variety of conditions, posing risks to food safety and human health. For example, Liu et al. (2010) showed that the *stx* gene was continuously expressed in VBNC *E. coli* O157:H7, which enabled the production of shiga toxins. In addition, serious food poisoning incidents caused by salted salmon roe contaminated with VBNC enterohemorrhagic *E. coli* O157:H7 were reported in Japan (Makino et al., 2000). Recently, VBNC *E. coli* O157:H7 in the phyllosphere of lettuce was also regarded as a food safety risk factor (Dinu and Bach, 2011). Therefore, elucidating the formation mechanism of VBNC *E. coli* O157:H7 will lay a theoretical foundation for preventing the outbreak of foodborne diseases caused by this pathogen.

Although the molecular mechanisms underlying the formation of the VBNC state of *E. coli* is not fully understood, several genes or proteins have been found to play an integral role in this process. A transcriptional regulator, RpoS, which regulates gene expression and enables bacteria to survive under various forms of environmental stress, was demonstrated to delay the entry of *E. coli* into the VBNC state (Boaretti et al., 2003). Outer membrane proteins (OMPs) are essential for the exchange of substances and responses to environmental stimuli in bacterial cells. Therefore, changes in the expression of OMPs, including OmpW and EnvZ, have a major impact on the survival of bacteria under stressful conditions, which was thought to be involved in the formation of VBNC *E. coli* (Asakura et al., 2008). In addition, ppGpp, regulated by the SpoT and RelA proteins, may be an inducer of the VBNC state (Chatterji et al., 1998). *E. coli* cells lacking ppGpp are less likely to enter the VBNC state than those that harbor this substance (Boaretti et al., 2003).

The formation of the VBNC state is closely related to cell culturability, but to date, few studies have reported the correlation between cell division and the formation of the VBNC state. Previously, our group performed a transcriptomic analysis of the HPCD-induced VBNC state of *E. coli* O157:H7 by RNA sequencing. The results showed that the expression of the *dicC* gene was downregulated by 20.08-fold in VBNC cells (Zhao et al., 2016). Nevertheless, how DicC regulates the formation of VBNC cells induced by HPCD remains unclear. In this study, the specific roles of cell division regulatory proteins (DicA and DicC) and the cell growth rate in VBNC *E. coli* O157:H7 formation were explored.

## Materials and Methods

### Bacterial Strains and Culture Conditions

The bacterial strains and plasmids used in this study are summarized in Table 1. *E. coli* O157:H7 NCTC 12900, a *stx*-negative strain, was used in this study and was obtained from the National Culture Type Collection (Colindale, London, United Kingdom). *E. coli* O157:H7 was cultured overnight in Luria-Bertani (LB) broth at 37°C (10 g/L tryptone, 5 g/L yeast extract, 10 g/L NaCl). The overnight culture was transferred to fresh LB broth at a dilution of 1:100 and inoculated with shaking (200 rpm) at 37°C to the exponential phase (OD_600_ = 0.8). LB agar was obtained by adding 1.5% (w/v) agar. When culturing the resistant strains or inducing protein expression, antibiotics or inducers were added into the culture as follows: ampicillin (100 μg/mL), kanamycin (25 μg/mL), arabinose (0.2% [w/v]), IPTG (1 mM).

**TABLE 1:** Bacterial strains and plasmids used in this study.

**Strain or plasmid**	**Characteristics**	**Source or reference**
**Strains**		
*Escherichia. coli*	The National Culture Type Collection (Colindale, London, United Kingdom).	
O157:H7 EDL933	Wild type; serotype O157:H7 NCTC 12900; Stx negative strain	
Δ*dicC*strain	O157:H7 EDL933 Δ*dicC*	This study
Δ*dicB* strain	O157:H7 EDL933 Δ*dicB*	This study
DicA strain	O157:H7 EDL933 strain containing pTHA-DicA	This study
DicC strain	O157:H7 EDL933 strain containing pTHA-DicC	This study
Vector strain	O157:H7 EDL933 containing pTrc-His A	This study
Δ*dicC*/DicC strain	Δ*dicC* strain containing pTHA-DicC	This study
*E. coli* DH5α	Used for cloning	TIANGEN BIOTECH
**Plasmids**		
pTrc-His A	Cloning vector; Km^r^	This study
pTHA-DicA	pTrc-His A with the DicA, fused with His tag at its N-terminal	This study
pTHA-DicC	pTrc-His A with the DicC, fused with His tag at its N-terminal	This study
pKD46	λ Red recombinase expression, which is induced by L-arabinose; Amp^r^	(Datsenko and Wanner, 2000)
pKD4	PCR template for Km cassette and FRT site; Amp^r^	(Datsenko and Wanner, 2000)
pCP20	Helper plasmid encoding recombinase; Amp^r^ and Cm^r^	(Datsenko and Wanner, 2000)

### Construction of Deletion Mutants and Complementary Strains

For the construction of the *dicC* deletion mutant, the ORF of DicC was replaced with a kanamycin resistance (Km^r^) gene cassette. The cassette was amplified from the pKD4 plasmid (obtained from the *E. coli* Genetic Stock Center, CGSC) by PCR using primer pairs containing an *E. coli* O157:H7 *dicC*-specific sequence (the primer sequences are listed in Table 2). Then, the pCP20 plasmid (Table 1) (Datsenko and Wanner, 2000), which can recognize the FRT sequence, was used to remove the Km^r^ cassette. Since the pCP20 plasmid was temperature-sensitive, it was removed by incubation at 42°C for 12 h. To construct the complemented strain of the Δ*dicC* mutant, the *dicC* gene amplified from the genomic DNA of wild-type (WT) strains was purified and cloned into pTrc-His A with the appropriate restriction sites for *Bam*HI and *Hin*dIII (New England Biolabs, Inc.) to form the pTHA-DicC plasmid, which was then transformed into the Δ*dicC* strain.

**TABLE 2:** Primers used in this study.

**Primers**	**Sequences (5′–3′)**	**Use**	**Length (bp)**
DicA-His-F	CGCGGATCCATGAAAAACGAAACCTTCGGTG	Amplification of DicA gene for complementation	408
DicA-His-R	CCCAAGCTTTTATTTATTTGCGCTTCTTTTGC		
DicC-His-F	CGCGGATCCATGTTGAAAATTGATGCTATAG	Amplification of DicC gene for complementation	228
DicC-His-R	CGGGGTACCTCAGTGGTGATTTTCATTGTTC		
Δ*dicC*-F	TTAGGTTTCTCTACGATCTAGTTTCCTTAGGAAAATC TAAGGGTTTCGATGATTGTGTAGGCTGGAGCTGCTTC	Amplification of Km cassette and FRT contain 50 bp of the *dicC* homologous sequence	795
Δ*dicC*-R	TCAGTGGTGATTTTCATTGTTCAACCGCCCC GCCCGCTTTGCCTTACGATGAATATCCTC CTTAGTTCCT ATTCC		
*dicA*-qF	GGAAAGAGATGAAACACAGCC	Real-time quantitative PCR amplification of cDNA from *E.coli* O157:H7 strain, its mutant and complementary strains with *dicA*, *dicB*, *dicC*, and *16S* gene	112
*dicA*-qR	TTCGCCTGGTTGCTTATC		
*dicB*-qF	CGCGGATCCATGGAAACGTTATTACCAAACG		189
*dicB*-qR	CCCAAGCTT TCATTGACGTTCTCCGAAAATAC		
*dicC*-qF	CAATGTCGCAGGAGTTAGG		126
*dicC*-qR	CCGCTTTGCCTTACGATA		
*16S*-qF	AGACCAAAGAGGGGGACCT		132
*16S*-qR	TTCCAGTGTGGCTGGTCAT		

*Note: the underlines represent cutting sites of restriction enzymes.*

### Recombinant Bacterial Strains Harboring Overexpression Plasmids for DicA and DicC

The full-length *dicC* and *dicA* genes were amplified by PCR with *E. coli* O157:H7 genomic DNA as the template using primer pairs (DicC-*Bam*HI and DicC-*Hin*dIII for *dicC*; DicA-*Bam*HI and DicA-*Hin*dIII for *dicA*) with restriction enzyme linkers (*Bam*HI and *Hin*dIII) (Table 2). The amplicons were digested with *Bam*HI and *Hin*dIII and then ligated to the vector pTrc-His A digested by the same enzymes and transformed into *E. coli* DH5α. pTHA-DicA and pTHA-DicC were harvested from the *E. coli* DH5α cells and transformed into the WT *E. coli* O157:H7 cells, which were further selected based on their kanamycin resistance. The full-length sequences of the *dicA* and *dicC* genes in the recombinant strains were verified by PCR and sequencing.

### Induction and Quantification of VBNC *E. coli* Cells

According to the experimental procedure previously described by Zhao et al. (2013), VBNC *E. coli* O157:H7 of the WT strain, mutant strains and recombinant strains were induced by HPCD at 5 MPa at 25°C for 40 min. These strains were also induced into the VBNC state under acid or oxidation treatment. The condition of acid induction was treatment with pH 2.5 sodium chloride (0.85% w/v) for 3 h, and the condition of oxidation induction was 50 mM H_2_O_2_ treatment for 6 h. The number of VBNC cells was determined by staining with the LIVE/DEAD *Bac*Light Bacterial Viability Kit (Molecular Probes, Inc., Eugene, OR, United States) in accordance with the manufacturer’s instructions and a previously published reference (Zhao et al., 2013), followed by analysis with a FACSCalibur flow cytometer (Becton Dickinson Immunocytometry Systems, San Jose, CA, United States). Furthermore, a Declipse C1 confocal laser scanning microscope (CLSM) was used for observation of stained cells. In addition, to determine the culturability of the cells treated with HPCD, the cells were pour-plated on LB agar in duplicate. When the culturable cell concentration was below 0.1 CFU/mL, the cells were considered to be in the VBNC state.

### Analysis of *dicA, dicB*, and *dicC* Gene Expression

The expression of the *dicA*, *dicB*, and *dicC* genes in the WT, recombinant and mutant strains was confirmed by real-time quantitative reverse transcription PCR (RT-qPCR). Briefly, cells were lysed, and RNA samples were extracted with the RNAprep Pure Cell/Bacteria Kit (TIANGEN BIOTECH, Beijing, China) according to the manufacturer’s instructions. RNA samples were reverse transcribed using the iScript^TM^ gDNA Clear cDNA Synthesis Kit (Bio-Rad, United States) according to the manufacturer’s instructions. Primers (Table 2) were designed using Beacon Designer 7.0 software (Premier Biosoft International, Palo Alto, CA, United States), and *16S* rRNA was used as the reference gene. Real-time PCR was performed using the CFX Connect^TM^ Real-Time PCR Detection System (Bio-Rad, United States) with ssoAdvanced^TM^ SYBR^®^ Green Supermix (Bio-Rad, United States). The relative expression of the genes was calculated using the 2^–Δ^ ^Δ^ ^Ct^ method (Livak and Schmittgen, 2001). Gene expression analyses were conducted in triplicate in at least two independent experiments.

### Total Protein Extraction and Western Blotting

The total proteins from the DicC- and DicA-overexpressing strains grown in LB broth at 37°C were extracted by suspending cells in SDS buffer [100 mM Tris-HCl (pH 6.8), 4% SDS, 20% glycerol, 0.2% bromophenol blue, 5% β-mercaptoethanol] and boiling for 10 min. The proteins were separated on 15% SDS-PAGE gels and then transferred onto nitrocellulose membranes. The membranes were blocked in TBST (Tris-buffered saline, 0.5% Tween-20) with 5% defatted milk, followed by incubation with primary anti-His antibody diluted 1:5,000 (GenScript Biotech, Corp., China) in TBST for 1 h at 37°C. The membranes were washed with TBST three times after incubation. Then, incubation with a horseradish peroxidase (HRP)-linked goat anti-rabbit secondary antibody (Bio-Rad, United States) diluted 1:3,000 in TBST was conducted at 37°C for 45 min. The bands of the target proteins in the membranes were detected by using an Amersham Imager 600 (GE Healthcare UK Ltd., Buckinghamshire, United Kingdom).

### Morphological Observation of the *ΔdicC* Mutant and Recombinant Strains

The morphology of the WT *E. coli* O157:H7 cells, Δ*dicC* mutants and recombinant strains was observed using scanning electron microscopy (SEM) and transmission electron microscopy (TEM) in accordance with the procedures described previously (Zhao et al., 2013). All the strains were grown to exponential phase before sample collection for SEM and TEM. For the TEM assay, the bacterial cells were examined on a JEM-1230 TEM instrument (JEOL Japan Electronics, Co., Ltd., Japan) at 80 kV at the Center of Biomedical Analysis, Tsinghua University (Beijing, China). For the SEM assay, observation and photomicrography were performed with a Hitachi S-3400 N SEM instrument (Hitachi Instruments, Inc., Japan) at the Center of Biomedical Analysis, Tsinghua University Center (Beijing, China). Data were recorded for at least six fields of view per strain. Figures in the results represent typical observations in each field. SEM images were used for the measurement of cell length through Image J 1.8.0 (National Institutes of Health, Bethesda, MD, United States). For each strain, cell length data were recorded from six fields of view, and at least 300 intact cells were measured.

### Statistical Analysis

Graphs were generated using Origin 9.2 software (Origin Lab Corporation, Northampton, MA, United States). Analysis of variance (ANOVA) was performed by IBM SPSS Statistics 20.0 (IBM Corporation, Armonk, NY, United States), and *p* < 0.05 was considered statistically significant for all assays.

## Results

### Construction of the *ΔdicC* Mutant Strain and DicA/DicC-Overexpressing Strains

**FIGURE 1:**
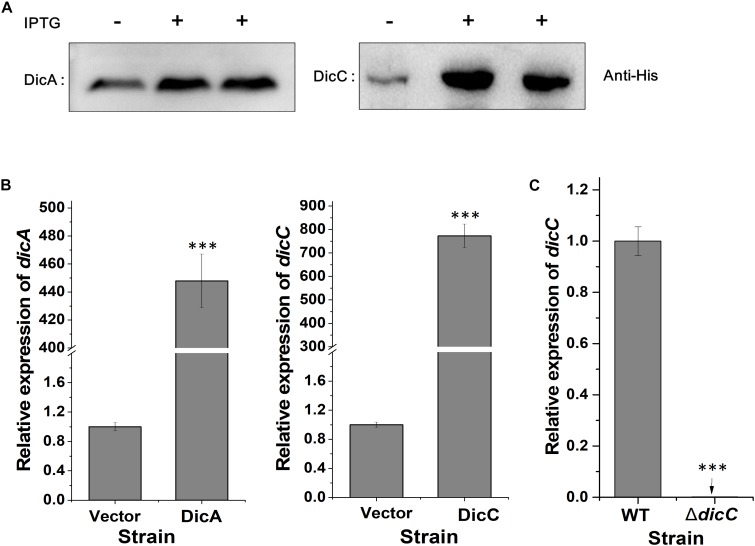
Construction of the DicA- and DicC-overexpressing and Δ*dicC* mutant strains. **(A)** Protein expression of DicA and DicC in recombinant strains induced by IPTG was confirmed by western blot analysis. **(B)** Detection of the *dicA* and *dicC* gene expression levels in the DicA and DicC recombinant strains by qPCR. **(C)** Detection of *dicC* expression in the Δ*dicC* mutant strain by qPCR. Error bars show the standard errors of the means. Significance was calculated by the *t*-test (^∗^*p* < 0.05, ^∗∗^*p* < 0.01, ^∗∗∗^*p* < 0.001).

Transcriptomic data from a previous study showed that the expression of the *dicC* gene was downregulated by 20.08-fold in VBNC *E. coli* O157:H7 cells induced by HPCD (Zhao et al., 2016). Here, to investigate whether deletion or overexpression of the *dicC* gene affects the population of HPCD-generated VBNC cells, a Δ*dicC* mutant strain and a DicC-overexpressing strain harboring the pTHA-DicC vector (DicC strain) were constructed. The DicC protein accumulation induced by IPTG in the DicC strain was verified by western blot analysis (Figure 1A). Compared with the vector strain transformed with the empty vector, the expression level of *dicC* increased dramatically in the DicC strain (Figure 1B). The Δ*dicC* mutant strain was obtained by homologous recombination, which was verified by detecting the expression level of *dicC* with qPCR (Figure 1C). In previous studies, it was found that *dicA* and *dicC* in bacteria simultaneously participated in the regulation of cell division (Béjar et al., 1986). Therefore, we also constructed a DicA-overexpressing recombinant strain (DicA strain). Compared with the vector strain, the mRNA and the protein levels of DicA strain were also significantly increased in the DicA strain (Figures 1A,B).

### Effects of DicC and DicA on the Formation of VBNC *E. coli* O157:H7 Induced by HPCD

**FIGURE 2:**
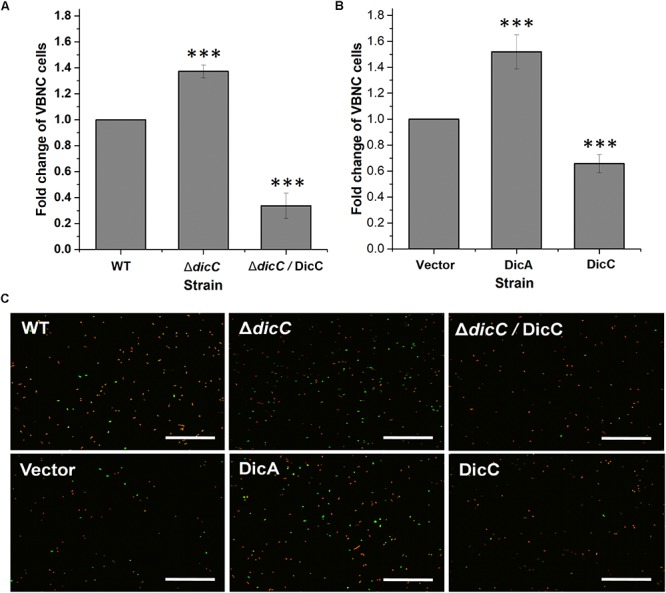
Effects of DicA and DicC on the percent changes in the number of VBNC cells induced by HPCD. **(A)** Changes in VBNC cell percentage generated by HPCD in the WT strain, Δ*dicC* mutant, and Δ*dicC*/DicC strain. **(B)** Changes in VBNC cell percentage generated by HPCD in the vector strain, DicC strain and DicA strain. **(C)** Photomicrographs of VBNC cells stained with the LIVE/DEAD BacLight Bacterial Viability Kit and examined under a fluorescence confocal microscope. Green: viable cells, red: dead cells. Bar = 20 μm. Error bars show the standard errors of the means. Significance was calculated by the *t*-test (^∗^*p* < 0.05, ^∗∗^*p* < 0.01, ^∗∗∗^*p* < 0.001).

Our previous study found that HPCD could induce the entry of *E. coli* O157:H7 into the VBNC state (Zhao et al., 2013). The number of VBNC cells of the WT, Δ*dicC* mutant and DicC complementary strains (Δ*dicC*/DicC strain) as well as the vector, DicC and DicA strains induced by HPCD were detected using the LIVE/DEAD *Bac*Light Bacterial Viability Kit followed by flow cytometry analysis. The results showed that compared with the WT strain, a larger number of Δ*dicC* mutant strain cells entered the VBNC state, while fewer VBNC cells were found in the Δ*dicC*/DicC strain (Figure 2A). Meanwhile, compared to the vector strain, the number of VBNC cells of the DicC strain decreased considerably upon HPCD induction (Figure 2B), which was consistent with our previous results (Zhao et al., 2016). These results suggested that DicC can negatively regulate the formation of VBNC cells induced by HPCD. Interestingly, we found that the number of VBNC cells formed in the DicA strain was higher than that in the vector strain (Figure 2B), which suggested that the DicA protein had the opposite effect on the regulation of the VBNC state, that is, DicA promoted the formation of VBNC cells. In addition, the number of VBNC cells was also observed directly by fluorescence confocal microscopy. The number of VBNC cells (green cells) in the DicC strain was significantly lower than that in the control strain, while the number of VBNC cells in the Δ*dicC* mutant strain was higher than that in the control strain, but decreasing again after DicC complementation in the mutant strain (Figure 2C). Similar to the above findings, the number of VBNC cells in the DicA strain was higher than that in the control strain (Figure 2C), indicating the role of DicC and DicA in the formation of the VBNC state. These results demonstrated that DicC negatively regulates the formation of VBNC *E. coli* O157:H7 induced by HPCD, while DicA could promote the process of VBNC cell formation.

### Regulatory Effect of DicC and DicA on the Growth Rate of *E. coli* O157:H7

**FIGURE 3:**
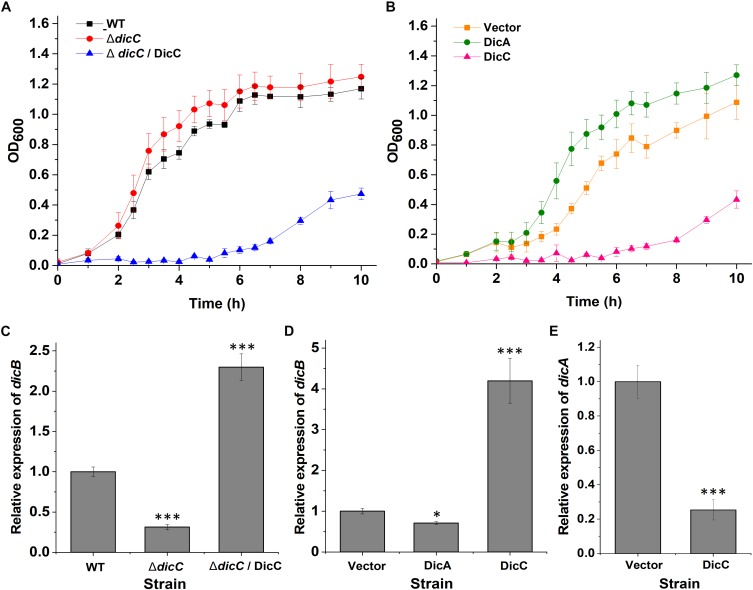
Effect of DicA and DicC on the growth rate of *E. coli* O157:H7. **(A)** Growth curves of the WT *E. coli* O157:H7, Δ*dicC* mutant and DicC complementary strains cultured at 37°C in LB. **(B)** Growth curves of the empty vector strain and DicA- and DicC-overexpressing strains cultured at 37°C in LB. **(C)** Detection of *dicB* gene expression in the WT, Δ*dicC* mutant and DicC complementary strains by qPCR. **(D)** Detection of *dicB* gene expression in the empty vector strain and DicA- and DicC-overexpressing strains by qPCR. **(E)** Relative expression of the *dicA* gene in the empty vector strain and DicC-overexpressing strain. Error bars show the standard errors of the means. Significance was calculated by the *t*-test (^∗^*p* < 0.05, ^∗∗^*p* < 0.01, ^∗∗∗^*p* < 0.001).

DicC and DicA have been shown to regulate cell division in bacteria, suggesting that these genes can affect the bacterial growth rate (Béjar and Bouché, 1985; Béjar et al., 1986; Yun et al., 2012). Therefore, the bacterial growth rates of the WT, Δ*dicC* mutant and Δ*dicC*/DicC complementary strains as well as the vector, DicC and DicA strains were tested. The experimental results showed that the Δ*dicC* mutant grew faster than the WT strain, while the Δ*dicC*/DicC complementary strain exhibited dramatically slower growth (Figure 3A), which may be due to the significantly high *dicC* expression level in the complementary strain. In addition, the growth rate of the DicC recombinant strain was obviously slower than that of the vector strain, but the cell growth rate increased in the DicA-overexpressing strain compared with the vector strain (Figure 3B). The above results indicated that DicC can inhibit bacterial cell division and reduce the growth rate, while DicA can promote cell division and enhance the growth rate.

It has been reported that DicC and DicA affect cell division and growth rate by regulating the expression of the *dicB* gene (Yun et al., 2012; Yang et al., 2016). Therefore, the expression of the *dicB* gene in the WT, mutant, vector and recombinant strains was determined by qPCR. The results showed that the transcription level of *dicB* was lower in the Δ*dicC* mutant strain and DicA strain than in the WT strain and vector strain, respectively, while it was significantly higher in the DicC recombinant strain than in the vector strain (Figures 3C,D). The different expression levels of *dicB* correlated with the differences in bacterial growth rates among the WT, Δ*dicC* mutant, vector, DicA recombinant and DicC recombinant strains. These results indicated that DicA could inhibit the expression of *dicB* and thereby promote cell division and increase the growth rate, while overexpression of DicC promoted the expression of the *dicB* gene and thereby inhibited cell division and decreased the growth rate.

To elucidate why overexpression of DicC promoted the expression of the *dicB* gene, we further tested the expression of the *dicA* gene in the DicC-overexpressing strain. Compared with the vector strain, the expression of the *dicA* gene was dramatically downregulated in the DicC-overexpressing strain (Figure 3E), which suggested that upregulation of *dicB* gene expression in the DicC-overexpressing strain was due to inhibition of *dicA* expression. These results indicated that overexpression of the *dicC* gene decreased the level of the *dicA* gene and that DicA plays a role in the inhibition of *dicB* gene expression. In this experiment, we found that the growth rates of the Δ*dicC* mutant and DicA strains increased, while that of the DicC strain decreased. Coincidentally, the ability of the two strains to form VBNC cells changed similarly compared with the control strains. These results suggest that changes in the bacterial growth rate may positively regulate the formation of VBNC cells. In addition, since DicA and DicC affect cell division by regulating *dicB*, we constructed Δ*dicB* mutant strain, and the experimental results showed that compared with the WT strain, the *dicB* gene was not expressed in the Δ*dicB* mutant. We also determined the number of VBNC cells formed in the Δ*dicB* mutant strain under HPCD treatment, and the results showed that, compared with the WT strain, the number of VBNC cells formed in the Δ*dicB* mutant strain increased significantly (Supplementary Figure S1). These results indicated that deletion of *dicB* could also promote VBNC formation.

### Morphological Changes in Cells of the *ΔdicC* Mutants and DicA/DicC-Overexpressing Strains

**FIGURE 4:**
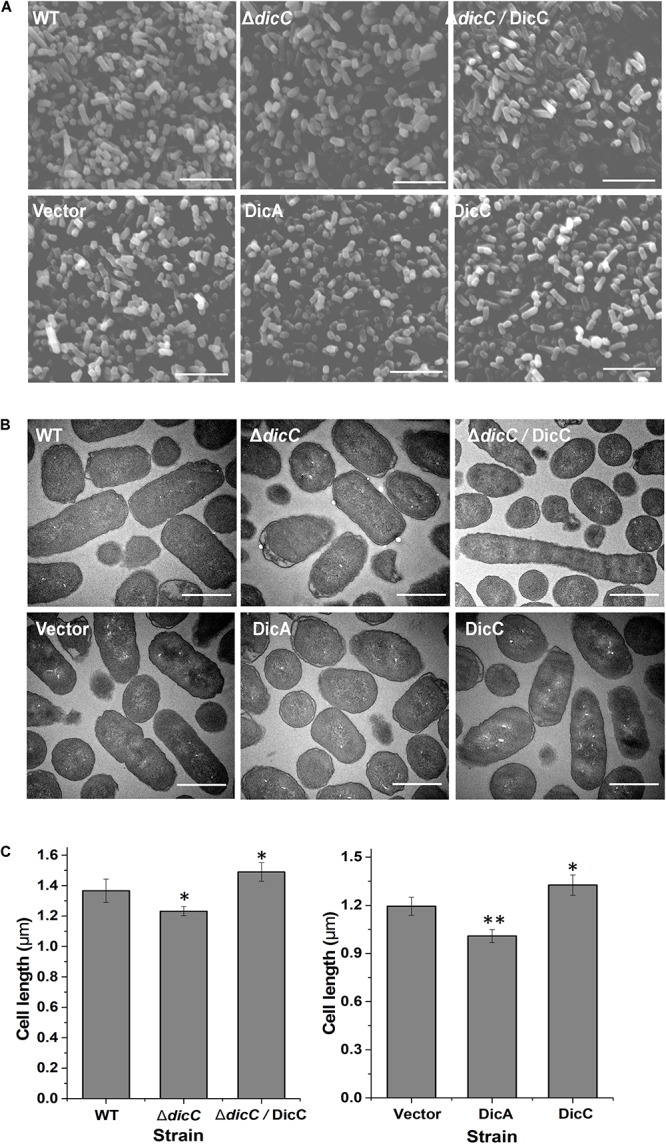
Changes in the cell morphology of the Δ*dicC* mutant train, Δ*dicC/*DicC complementary strain and *dicA*, *dicC-*overexpressing strain. **(A)** Scanning electron microscopy (SEM) (magnification of × 15,000). **(B)** Transmission electron microscopy (TEM) (magnification of × 15,000) photomicrographs. **(C)** Cell length statistical data of the WT, Δ*dicC* mutant, Δ*dicC/*DicC complementary, vector, DicA, and DicC strains. Bar = 5 μm **(A)**, bar = 1 μm **(B)**. SEM images were used for the measurement of cell length. For each strain, cell length data were recorded from six fields of view, and at least 300 intact cells were measured. Significance was calculated by the *t*-test (^∗^*p* < 0.05, ^∗∗^*p* < 0.01, ^∗∗∗^*p* < 0.001).

Changes in cell morphology are related to the adaptation of bacteria to external adversities (Justice et al., 2008). Many bacterial cell morphological changes occur after entry into the VBNC state (Chaiyanan et al., 2007; Oh et al., 2015; Zhao et al., 2017). One of the most important characteristics is that the cells exhibit dwarfism or shift from bacilli to cocci when entering the VBNC state, which helps bacterial cells better cope with external stress conditions. However, it is rarely studied whether the changes in bacterial cell morphology affect the formation of the VBNC state. Therefore, we observed the cell morphology of the Δ*dicC* mutant and DicC-overexpressing recombinant strains by SEM and TEM. The results showed that the cells of the Δ*dicC* mutant strain were shorter and rounder than those of the WT strain, while the cells of the Δ*dicC*/DicC complementary strain were longer (Figures 4A,B, top). The cell morphologies of the vector strain, DicC strain, and DicA strain were also observed. The cells of the DicC strain were much longer and the cells of the DicA strain were rounder and shorter than vector strain (Figures 4A,B, bottom). Furthermore, the cell lengths of the strains were recorded. The results showed that the cell lengths of the Δ*dicC* mutant strain and the DicA-overexpressing strain were significantly lower than those of the WT strain and the empty vector strain, respectively (Figure 4C). The cell lengths of the DicC complementary strain and DicC-overexpressing strain were higher than those of the WT strain and empty vector strain, respectively (Figure 4C). These results further indicated that the *dicC* and *dicA* genes had an impact on the cell length of *E. coli* O157:H7 cells. The different morphologies of the Δ*dicC* mutant and DicC/DicA-overexpressing strains may also be responsible for the different abilities of the strains to form VBNC cells. Therefore, these results suggested that the different morphologies of bacteria may affect their entry into the VBNC state.

### Impacts of DicC and DicA on the Formation of VBNC *E. coli* O157:H7 Cells Under Acid and H_2_O_2_ Stress

**FIGURE 5:**
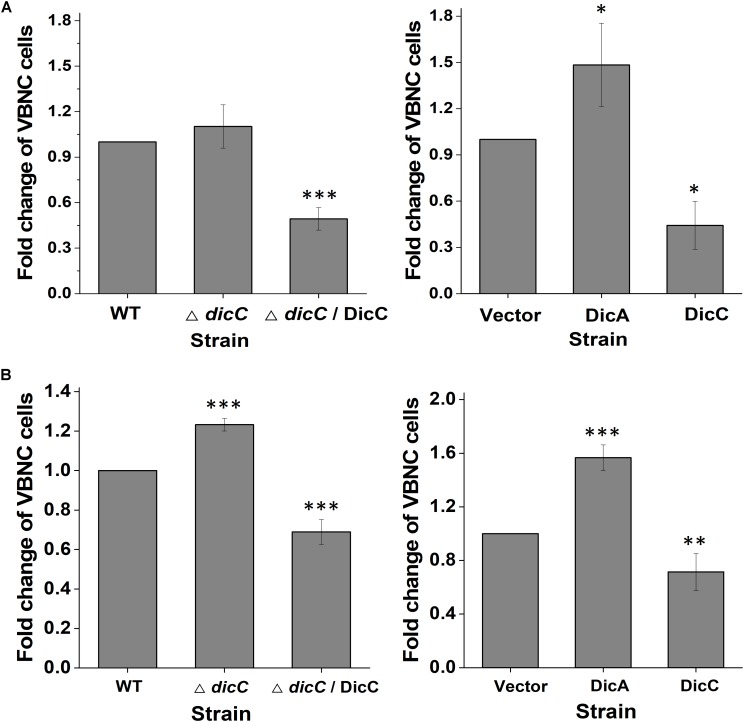
Effects of DicC and DicA on the ability of *E. coli* O157:H7 to form the VBNC state under acid stress **(A)** and H_2_O_2_ stress **(B). (A)** Changes in the VBNC cell percentage in the WT, Δ*dicC* mutant, Δ*dicC*/DicC, vector, DicC strain and DicA strains under pH 2.5 sodium chloride. **(B)** Changes in the VBNC cell percentage in the WT, Δ*dicC* mutant, and Δ*dicC*/DicC, vector, DicC and DicA strains under 50 mM H_2_O_2_. Error bars show the standard errors of the means. Significance was calculated by the *t*-test (^∗^*p* < 0.05, ^∗∗^*p* < 0.01, ^∗∗∗^*p* < 0.001).

Previous studies have shown that *E. coli* can enter the VBNC state under different adverse conditions, including acid and oxidative stress (Ding et al., 2016). To confirm whether the DicC and DicA proteins influence the ability of *E. coli* to form a VBNC state under other adverse conditions, *E. coli* O157:H7 in the exponential growth phase was treated with pH 2.5 sodium chloride (0.85% w/v) and 50 mM H_2_O_2_. The results showed that compared with the WT strain, the proportion of Δ*dicC* mutant strain cells entering the VBNC state was higher under acid and H_2_O_2_ stress, while the proportion was significantly lower for the Δ*dicC*/DicC complementary strain (Figures 5A,B). We also tested the ability of DicC- and DicA-overexpressing strains to form VBNC cells under acid and H_2_O_2_ stress. The results showed that the ability of the DicC-overexpressing recombinant strain to form VBNC state under acid and H_2_O_2_ stress was poorer than that of the vector strain, but the ability of the DicA-overexpressing strain was stronger than that of the vector strain (Figures 5A,B). Meanwhile, compared with the WT strain, the number of VBNC cells formed in the Δ*dicB* mutants increased significantly under acidic and oxidative conditions (Supplementary Figure S1). These results indicated that similar to HPCD induction, DicC can also delay the formation of VBNC *E. coli* cells, and DicA can promote this process under acid and H_2_O_2_ stress, suggesting that the regulation of cell division and growth rate by DicC may also be positively correlated with bacterial VBNC state formation under acidic and oxidative stress.

## Discussion

Our previous transcriptomic and proteomic study suggested that DicC might be involved in the formation of the VBNC state of *E. coli* O157:H7 induced by HPCD (Zhao et al., 2013). In this study, the specific role of DicC and DicA, as well as the association with cell growth rate and cell morphology in the formation of VBNC *E. coli* O157:H7, was demonstrated through gene deletion and overexpression. DicC delays the formation of the VBNC state, while DicA facilitates the formation of the VBNC state.

In *E. coli*, phage-encoded genes play an important role in host survival under adverse environmental conditions (Brussow et al., 2004; Wang et al., 2010). The Dic family proteins of *E. coli* encoded by the Kim (Qin) phage, one of nine cryptic prophages in *E. coli*, contains three genes encoding the DicA, DicB and DicC proteins and one sRNA transcribed by DicF (Cam et al., 1988; Yang et al., 2016). FtsZ, a bacterial cytoskeletal protein, plays a key role in bacterial cell division, which is spatially regulated by Min oscillation through inhibition of Z ring formation (Zhuo et al., 2007; Erickson et al., 2010). Nevertheless, the DicB protein can enhance the division-inhibitory activity of MinC, which is associated with Min oscillation and can restrain the division of bacterial cells (Johnson et al., 2002). Under normal circumstances, the expression of the *dicB* gene in cells is relatively low, and cells can divide normally; however, when the expression of the *dicB* gene is activated, cell division rapidly ceases (Labie et al., 1989). A previous study indicated that DicA can inhibit the transcription of the *dicB* gene (Béjar et al., 1988). In addition, recent advances indicated that downregulation of the *dicA* gene by CnuK9E, a mutant of the OriC-binding nucleoid protein (Cnu), could induce a burst of *dicB* expression and inhibit cell division (Yun et al., 2012). In this study, we found that overexpression of the *dicA* gene resulted in an increase in the cell growth rate, implying that the expression levels of *dicB* decreased and cell division was suppressed, which is consistent with previous studies.

Relevant studies on the function of *dicC* gene are relatively rare. Previous studies have shown that the DicC protein can complement the function of the DicA protein, thereby inhibiting the expression of the *dicB* gene, but the evidence is unclear (Béjar et al., 1986). In this study, we observed the opposite phenomenon: when *dicC* was overexpressed, the cell growth rate of the DicC strain decreased. In the Δ*dicC* mutants, the expression level of the *dicB* gene decreased, and the cell growth rate became higher than that in the WT strain. To further explain this phenomenon, the regulatory relationship between the *dicA* and *dicC* genes was demonstrated. It was demonstrated for the first time that the expression level of the *dicB* gene increased and that *dicA* was downregulated in the DicC-overexpressing strain, which suggested that the increased expression of *dicB* was due to the inhibition of *dicA*. This finding also indirectly indicated that *dicA* plays a major role in inhibiting the expression of *dicB*. In addition, the results also implied that DicC might inhibit the expression of the *dicA* gene to some extent, possibly because these two genes share a common promoter region, but the direction of transcription is the opposite. Yun et al. (2012) found that overexpression of the *dicA* gene could significantly inhibit the expression of *dicC*, which was consistent with the results of this study. Here, we confirmed that overexpression of *dicC* can also inhibit the expression of DicA, thereby initiating *dicB* transcription and finally inhibiting bacterial growth. The construction of the *dicA* deletion mutant was not successful in this study, which may be due to the deletion of *dicA* leading to significantly increased expression of *dicB* and bacterial growth was suppressed, so positive bacterial colonies could not be screened.

To date, there have been some reports regarding the relationship between cell division and VBNC formation; however, these studies were conducted at the omics level, while few studies regarding specific mechanisms have been reported. Xu et al. (2018) showed that the expression of cell division-related genes, including *ftsZ, ftsA, ftsQ*, and *ftsH*, was downregulated in VBNC *V. cholerae*, while *minC* and *slmA* were upregulated, leading to inhibition of cell division. Rozen et al. (2002) reported that the suppressed genes were related to cell division and suggested mechanisms for the cessation of growth in VBNC *E. coli*. In addition to these omics studies, a recent study found that the *cpdA* gene, encoding cAMP phosphodiesterase, delayed the formation of the *E. coli* VBNC state by reducing intracellular cAMP levels, which negatively regulated cell division and growth under stress conditions. That is, the lack of cAMP-CRP effectively led to retention of high colony forming activity and promoted cell division, which was not conducive to the formation of the VBNC state (Nosho et al., 2018). These results suggested that the growth rate of *E. coli* cells was negatively correlated with VBNC formation. Contrary to the results in this study, our results indicated that the bacterial growth rate was positively correlated with the formation of the VBNC state. A previous report proposed that VBNC state may be part of a dormant state in which active cells exist stochastically (Ayrapetyan et al., 2015b). Interestingly, studies have also shown that the VBNC state exists stochastically in unstressed growing cultures, similar to the characteristics of persister cells (Orman and Brynildsen, 2013; Ayrapetyan et al., 2015a). It was found that even during the logarithmic phase, there were more viable cells than culturable cells of *Vibrio vulnificus*, suggesting that VBNC cells existed stochastically during logarithmic-phase growth (Ayrapetyan et al., 2015a). Therefore, we speculate that in this study, with the increase in the cell growth rate of the DicA-overexpressing strain and the Δ*dicC* mutant strain, the number of VBNC cells randomly generated during logarithmic growth may also increase, eventually leading to an increase in the number of VBNC cells induced by stress; however, this hypothesis needs to be further verified.

Changes in cell morphology and cell size are distinct characteristics of VBNC cells (Zhao et al., 2017). Compared with culturable cells, VBNC cells may exhibit dwarfism. This condition provides the cells with increased surface area for nutrient uptake, which is beneficial for cell survival under stressful conditions such as starvation, low temperature and extreme pH (Zhao et al., 2017). Gupte et al. (2003) observed that *Salmonella typhi* cells exhibited gradual rounding after entering the VBNC state. The change from a bacillary to a coccoid form was also observed in VBNC *Vibrio parahaemolyticus*, with the occurrence of wrinkles and herpes-like structures on the cell surface (Chen et al., 2009). Similar structures were reported in VBNC *E. coli* cells, accompanied by enriched intracellular DNA (Signoretto et al., 2002; Sachidanandham et al., 2005). In addition, the shapes of *Campylobacter jejuni* and *Edwardsiella tarda* were found to transform from the spiral to coccoid form and from short rods to the coccoid form, respectively (Chaisowwong et al., 2012). Currently, the study of morphological changes in VBNC cells is limited to the description of characteristics, but there is no evidence regarding whether the changes affect the formation of VBNC cells. In this study, it was found that after deletion of the *dicC* gene and overexpression of the *dicA* gene, an increase in bacterial growth rate was accompanied by the formation of a short and round shape compared with the WT strain, which was similar to the morphology of VBNC bacteria. Moreover, an increased number of cells of the two strains entered the VBNC state after induction by different stress factors. Hence, these results indirectly indicated that changes in morphology could affect bacterial entry into the VBNC state. It was presented for the first time that a short and round shape might be favorable for bacterial entry into the VBNC state.

## Conclusion

This study proved for the first time that the formation of the VBNC state induced by HPCD was associated with the cell division-related genes *dicC* and *dicA*. Our investigation demonstrated that the cell growth rate regulated by DicC and DicA with changes in cell morphology may be positively correlated with the formation of the VBNC state. Elucidation of the molecular mechanism of the VBNC state will provide insights into bacterial stress responses and the physiological characteristics of the VBNC state, which may provide inspiration for the development of novel detection methods for VBNC bacteria or direct prevention of the entry of cells into this hard-to-detect state.

## Data Availability Statement

The datasets generated for this study are available on request to the corresponding author.

## Author Contributions

HP and KD carried out the experiments and wrote the manuscript. LR gave advice and assistance during the experiments. YW and LZ designed the experiments and revised the manuscript. XL revised the manuscript.

## Conflict of Interest

The authors declare that the research was conducted in the absence of any commercial or financial relationships that could be construed as a potential conflict of interest.
