# Supplementary material for: The Association of Cell Division Regulated by DicC With the Formation of Viable but Non-culturable Escherichia coli O157:H7
Source: Front Microbiol. 2019 Dec 10;10:2850. doi: 10.3389/fmicb.2019.02850 (PMC6915034; doi:10.3389/fmicb.2019.02850)
Supplement: Supplementary file 1 [file Image_1.pdf]

Supplementary Material

1.1 Supplementary Figures

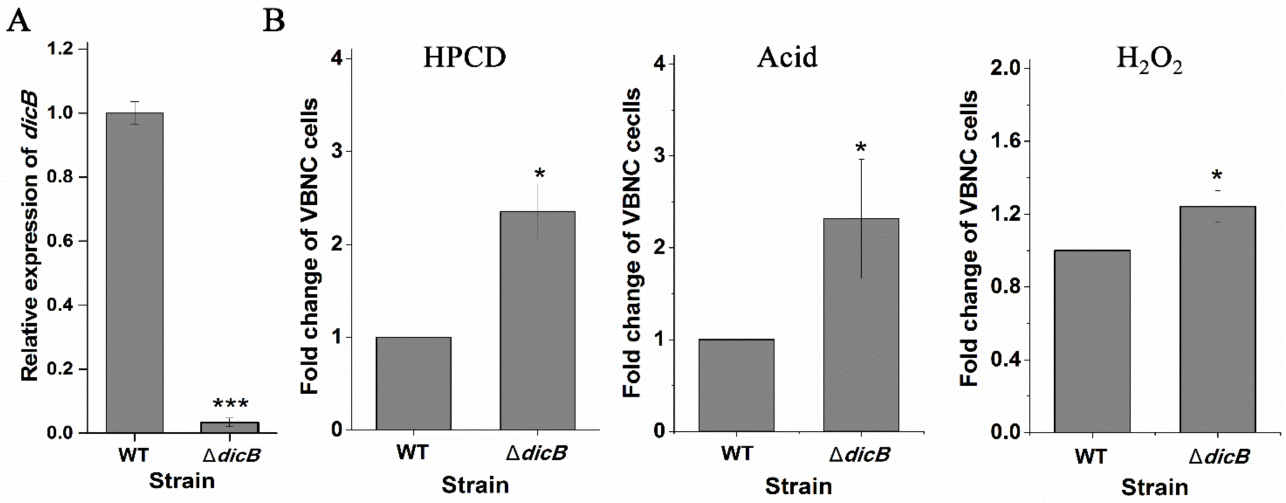

Supplementary Figure 1. Fold changes in the number of VBNC cells induced by HPCD, acid, and H_2O_2 stress in the WT and Δ*dicB* mutant strains. (A) Detection of *dicB* gene expression in the WT strain and Δ*dicB* mutant strain by qPCR. **(B)** Changes in the VBNC cell percentage in the WT and Δ*dicB* mutant strains under HPCD, acid, and H_2O_2 stress. Error bars show the standard errors of the means. Significance was calculated by the t-test (* $p < 0.05$, ** $p < 0.01$).
